# Supplementary material for: Cortical Bone Loss and Fragility in a 2-Month Triple Transgenic Mouse Model of Alzheimer’s Disease
Source: Cells. 2025 Nov 19;14(22):1816. doi: 10.3390/cells14221816 (PMC12650978; doi:10.3390/cells14221816)
Supplement: Supplementary file 1 [file cells-14-01816-s001.zip › cells-3949660-supplementary.pdf]

Cells
Supporting Information for

Cortical Bone Loss and Fragility Precedes Amyloid Deposition in a 2-month Triple Transgenic Mouse Model of Alzheimer's Disease

Giuseppina Storlino[1#], Francesca Posa[1#], Teresa Stefania Dell'Endice[1], Federica Piccolo[1], Graziana Colaianni[2], Tommaso Cassano[3], Maria Grano[2], Giorgio Mori[1*].

**Mice Id**	**Old**	**Sex**	**Total Area (T.Ar) (mm^2)**	**Bone Area (B.Ar) (mm^2)**	**Periosteal Perimeter (P.Pm) (mm)**	**Endosteal Perimeter (E.Pm) (mm)**	**Polar Moment of Inertia (p.MOI) (mm^4)**
non-Tg_1	2-months	male	2,3553	0,9162	11,588	6,1732	0,61315
non-Tg_2	2-months	male	2,2846	0,88955	10,826	5,8178	0,54571
non-Tg_3	2-months	male	1,7754	0,75671	9,4068	5,1201	0,35048
non-Tg_4	2-months	male	2,0314	0,81411	10,1	5,5555	0,44222
non-Tg_5	2-months	male	1,71	0,75849	8,8062	4,9987	0,33059
non-Tg_6	2-months	male	1,7865	0,8303	9,0486	5,0967	0,36325
non-Tg_7	2-months	male	1,6979	0,74032	8,8588	4,965	0,31822
non-Tg_8	2-months	male	2,2997	*	11,349	5,9284	0,67715
non-Tg_9	2-months	male	2,3246	*	10,97	5,8937	0,61559
Tg_1	2-months	male	1,2183	0,53681	7,409	4,2129	0,16663
Tg_2	2-months	male	1,5437	0,65935	8,4272	4,7351	0,26303
Tg_3	2-months	male	1,7129	0,74494	9,0845	4,971	0,32456
Tg_4	2-months	male	1,7789	0,72462	9,0524	5,0575	0,32997
Tg_5	2-months	male	1,6873	0,71256	8,9487	5,0125	0,30935
Tg_6	2-months	male	1,6171	0,65041	8,7627	4,8289	0,27241
Tg_7	2-months	male	1,7555	0,78115	9,3174	5,0766	0,35334
Tg_8	2-months	male	*	*	*	*	*
Tg_9	2-months	male	*	*	*	*	*

Table S1 Femur Cortical Micro-CT Data. Data obtained using Bruker Skyscan 1276 version 1.8 and reconstructions and analyses performed using TC Skyscan analyzer (CTAn version 1.20.8.0). * Value could not be obtained.

Mice Id	Old	Sex	Total Area (T.Ar) (mm^2)	Bone Area (B.Ar) (mm^2)	Periosteal Perimeter (P.Pm) (mm)	Endosteal Perimeter (E.Pm) (mm)	Polar Moment of Inertia (p.MOI) (mm^4)
non-Tg_1	2-months	male	1,21	1,18	11,6	10,6	0,732
non-Tg_2	2-months	male	1,28	1,25	11,6	10,7	0,791
non-Tg_3	2-months	male	1,15	1,14	10,1	9,49	0,586
non-Tg_4	2-months	male	1,18	1,17	10,7	10,2	0,673
non-Tg_5	2-months	male	0,909	0,896	*	*	*
non-Tg_6	2-months	male	1,11	1,1	9,98	9,64	0,546
non-Tg_7	2-months	male	1,04	1,03	9,65	9,29	0,488
non-Tg_8	2-months	male	1,34	1,32	10,3	9,83	0,732
non-Tg_9	2-months	male	1,24	1,23	10,5	10,1	0,67
Tg_1	2-months	male	0,725	0,719	7,05	6,84	0,202
Tg_2	2-months	male	0,846	0,836	7,55	7,28	0,25
Tg_3	2-months	male	1,02	1,01	8,65	8,3	0,42
Tg_4	2-months	male	0,798	0,791	7,48	7,2	0,231
Tg_5	2-months	male	1,03	1,02	8,93	8,62	0,448
Tg_6	2-months	male	0,962	0,95	9,07	8,66	0,414
Tg_7	2-months	male	1,08	1,07	8,93	8,49	0,462
Tg_8	2-months	male	1,25	1,23	10,3	9,59	*
Tg_9	2-months	male	1,08	1,06	8,57	7,91	0,378

Table S2 Tibiae Cortical Micro-CT Data. Data obtained using Bruker Skyscan 1276 version 1.8 and reconstructions and analyses performed using TC Skyscan analyzer (CTAn version 1.20.8.0). * Value could not be obtained.

			Femur		Tibia	
**Mice Id**	**Old**	**Sex**	**Maximum Load to Fracture (N)**	**Stiffness (N/mm)**	**Maximum Load to Fracture (N)**	**Stiffness (N/mm)**
non-Tg_1	2-months	male	19,89898	2,717708	12,19283	1,813747
non-Tg_2	2-months	male	21,29372	2,81663	18,20273	2,407769
non-Tg_3	2-months	male	18,30237	2,420948	20,28206	2,757885
non-Tg_4	2-months	male	18,76854	2,482611	19,5414	2,699202
non-Tg_5	2-months	male	19,32395	2,556078	7,751636	*
non-Tg_6	2-months	male	23,0431	3,233732	21,63439	2,861692
non-Tg_7	2-months	male	20,87667	2,795139	15,22955	2,014491
non-Tg_8	2-months	male	28,26483	3,85475	9,607151	2,013915
non-Tg_9	2-months	male	24,83878	3,285553	16,4643	2,177818
Tg_1	2-months	male	13,16548	1,741466	11,22969	1,496957
Tg_2	2-months	male	17,864	2,362964	11,36097	1,502773
Tg_3	2-months	male	9,896203	2,255406	19,96476	2,640841
Tg_4	2-months	male	19,36761	2,561854	10,08977	1,334625
Tg_5	2-months	male	22,4899	2,974855	8,181358	1,08219
Tg_6	2-months	male	19,38002	2,732582	17,37289	2,302604
Tg_7	2-months	male	19,65755	2,677685	11,33312	1,507531
Tg_8	2-months	male	*	*	22,18841	*
Tg_9	2-months	male	*	*	25,95181	*

Table S3 Femur and Tibia 3-Point Bending Test Data. Data obtained using Zwick tensile testing machine (ZwickiLine Z1.0). The software used to control the machine and for data acquisition and analysis was Zwich/Roell testXpert III version 1.4. * Value could not be obtained.

Mice Id	Old	Sex	% OYs Empty Lacunae	% TRAP+ OYs/Total Lacunae	% Ct. Nr. OCs/BS	% Tb. Nr. OCs/BS	% Ct. TRAP Surface/BS	% Tb. TRAP Surface/BS
non-Tg_1	2-months	male	8,29026	19,96802	0,00070	0,07557	0,04269	6,93689
non-Tg_2	2-months	male	7,68483	15,14286	0,00590	0,10074	0,51967	8,23964
non-Tg_3	2-months	male	11,68242	12,93333	0,00220	0,02184	0,19252	2,10748
non-Tg_4	2-months	male	12,50229	8,73189	0,00148	0,05196	0,07147	3,02239
non-Tg_5	2-months	male	13,58623	15,12477	0,00849	0,04966	0,81297	1,76785
non-Tg_6	2-months	male	11,37841	*	0,00333	*	0,26943	*
non-Tg_7	2-months	male	*	*	*	*	0,48684	*
non-Tg_8	2-months	male	*	*	*	*	*	*
non-Tg_9	2-months	male	*	*	*	*	*	*
Tg_1	2-months	male	11,43819	11,67532	0,00921	0,04479	0,75260	2,82240
Tg_2	2-months	male	17,70382	13,29231	0,00761	0,06974	0,82943	4,12325
Tg_3	2-months	male	19,94047	7,75350	0,00427	0,06942	0,27525	3,62289
Tg_4	2-months	male	17,23520	11,58662	0,00769	0,06452	1,22303	7,28053
Tg_5	2-months	male	12,57062	7,76738	0,00579	0,10464	0,68047	5,49125
Tg_6	2-months	male	*	5,64103	0,00457	0,06844	0,73939	4,82297
Tg_7	2-months	male	*	9,93459	0,01070	0,14162	1,28477	3,81368
Tg_8	2-months	male	*	*	*	0,06149	0,42807	2,41310
Tg_9	2-months	male	*	*	*	*	*	*

Table S4 Histological Analysis Data. Data obtained using Hematoxylin and Eosin stains for %OYs Empty Lacunae (scale bars 20 mm); TRAP stains for %TRAP+ OYs/Total Lacunae, % Ct. Nr. OCs/BS, %Tb. Nr. OCs/BS, %Ct. TRAP Surface/BS, and %Tb. TRAP Surface/BS (Scale bars 25 mm). The images were viewed under a microscope Nikon Eclipse Ts2R (Nikon, Minato, Tokyo, Japan) and acquired with a 20/40x objective lens. The analysis was performed using ImageJ software version 1.5. * Value could not be obtained.

Mice Id	Old	Sex	Bone Mineral Density (BMD) (gHA/cm^3)	Bone Volume/Total Volume (BV/TV) (%)	Degree of Anisotropy (DA)	Fractal Dimension (FD)	Trabecular Number (Tb.N) (1/mm)	Trabecular Separation (Tb.Sp) (mm)	Trabecular Thickness (Tb.Th) (mm)
non-Tg_1	2-months	male	0,3250	17,100	1,590	2,220	3,290	0,177	0,052
non-Tg_2	2-months	male	0,3260	16,100	1,380	2,180	3,490	0,184	0,046
non-Tg_3	2-months	male	0,3480	17,600	1,260	2,350	3,460	0,179	0,051
non-Tg_4	2-months	male	0,2940	12,400	1,780	2,260	2,550	0,224	0,049
non-Tg_5	2-months	male	0,2030	3,630	1,310	1,970	*	0,355	0,050
non-Tg_6	2-months	male	0,3220	14,200	1,170	2,340	2,700	0,198	0,053
non-Tg_7	2-months	male	0,2660	9,320	1,370	2,200	1,580	0,285	0,059
non-Tg_8	2-months	male	0,3880	19,800	1,620	2,370	3,130	0,196	0,063
non-Tg_9	2-months	male	0,3700	17,700	1,820	2,380	2,840	0,207	0,062
Tg_1	2-months	male	0,3180	15,700	1,530	2,280	3,160	0,191	0,050
Tg_2	2-months	male	0,3650	19,800	1,460	2,330	3,520	0,179	0,056
Tg_3	2-months	male	0,3740	20,700	1,100	2,350	3,550	0,172	0,058
Tg_4	2-months	male	0,2860	13,300	1,610	2,150	2,540	0,214	0,052
Tg_5	2-months	male	0,2300	11,500	1,930	2,160	2,240	0,231	0,051
Tg_6	2-months	male	0,2390	12,300	1,620	2,100	2,380	0,19	0,052
Tg_7	2-months	male	0,2980	15,600	1,290	2,300	3,080	0,197	0,051
Tg_8	2-months	male	0,4490	28,500	1,350	2,490	5,140	0,138	0,056
Tg_9	2-months	male	0,4370	25,000	1,470	2,440	4,210	0,168	0,059

Table S5 Femur Trabecular Micro-CT Data. Data obtained using Bruker Skyscan 1276 version 1.8 and reconstructions and analyses performed using TC Skyscan analyzer (CTAn version 1.20.8.0). * Value could not be obtained.

Mice Id	Old	Sex	Bone Mineral Density (BMD) (gHA/cm^3)	Bone Volume/Total Volume (BV/TV) (%)	Degree of Anisotropy (DA)	Fractal Dimension (FD)	Trabecular Number (Tb.N) (1/mm)	Trabecular Separation (Tb.Sp) (mm)	Trabecular Thickness (Tb.Th) (mm)
non-Tg_1	2-months	male	0,306	14,100	2,200	2,150	2,760	0,198	0,051
non-Tg_2	2-months	male	0,273	10,800	2,420	2,050	2,120	0,306	0,051
non-Tg_3	2-months	male	0,343	16,700	1,900	2,300	3,310	0,178	0,050
non-Tg_4	2-months	male	0,308	13,000	1,930	2,240	2,690	0,204	0,048
non-Tg_5	2-months	male	0,226	13,700	1,930	1,930	*	0,418	0,064
non-Tg_6	2-months	male	0,314	13,600	2,100	2,250	2,470	0,226	0,055
non-Tg_7	2-months	male	0,259	*	1,230	2,070	1,640	0,284	0,053
non-Tg_8	2-months	male	0,326	12,900	2,370	2,140	2,120	0,263	0,061
non-Tg_9	2-months	male	0,345	14,900	1,930	2,240	2,550	0,222	0,058
Tg_1	2-months	male	0,337	16,900	2,240	2,150	3,200	0,221	0,053
Tg_2	2-months	male	0,355	18,400	1,570	2,210	3,340	0,199	0,055
Tg_3	2-months	male	0,339	18,100	1,670	2,210	2,880	0,260	0,063
Tg_4	2-months	male	0,293	13,000	1,870	2,120	2,410	0,252	0,054
Tg_5	2-months	male	0,314	14,700	2,170	2,140	2,510	0,233	0,058
Tg_6	2-months	male	0,316	16,200	1,510	2,250	3,160	0,203	0,051
Tg_7	2-months	male	0,27	9,920	2,040	1,980	2,130	0,327	0,047
Tg_8	2-months	male	0,411	*	2,880	2,330	4,240	0,169	0,053
Tg_9	2-months	male	0,373	18,200	3,180	2,270	3,340	0,206	0,055

Table S6 Tibiae Trabecular Micro-CT Data. Data obtained using Bruker Skyscan 1276 version 1.8 and reconstructions and analyses performed using TC Skyscan analyzer (CTAn version 1.20.8.0). * Value could not be obtained.

Parameters	non-Tg	3xTg-AD
T.Ar (femur)	non normal distribution	normal
B.Ar (femur)	normal	normal
P.Pm (femur)	normal	normal
E.Pm (femur)	normal	normal
p.MOI (femur)	normal	normal
T.Ar (tibiae)	normal	normal
B.Ar (tibiae)	normal	normal
P.Pm (tibiae)	normal	normal
E.Pm (tibiae)	normal	normal
p.MOI (tibiae)	normal	normal
Tb. BMD (femur)	normal	normal
BV/TV (femur)	normal	normal
DA (femur)	normal	normal
FD (femur)	normal	normal
Tb.N (femur)	normal	normal
Tb.Sp (femur)	non normal distribution	normal
Tb.Th (femur)	normal	normal
Tb. BMD (tibiae)	normal	normal
BV/TV (tibiae)	normal	normal
DA (tibiae)	normal	normal
FD (tibiae)	normal	normal
Tb.N (tibiae)	normal	normal
Tb.Sp (tibiae)	normal	normal
Tb.Th (tibiae)	normal	normal
Maximum Load to Fracture (femur)	normal	normal
Stiffness (femur)	normal	normal
Maximum Load to Fracture (tibiae)	normal	normal
Stiffness (tibiae)	normal	normal
%OYs Empty Lacunae	normal	normal
%TRAP+ OYs/ Tot Lacunae	normal	normal
%Ct. Nr. OCs/BS	normal	normal
%Tb. Nr. OCs/BS	normal	non normal distribution
%Ct. TRAP Surface/BS	normal	normal
%Tb. TRAP Surface/BS	normal	normal

Table S7 Data distribution. The Shapiro-Wilk normality test was used to assess the distribution of the samples Samples with normal distribution were compared using the Welch-corrected t-test, while those with non-normal distribution were compared using the Mann-Whitney test.

Bone parameters Fig.1	Comparison between groups	p value	Choen's d
T.Ar	non-Tg vs 3xTg-AD	**p=0.0079	1.681
B.Ar	non-Tg vs 3xTg-AD	**p=0.0079	1.711
P.Pm	non-Tg vs 3xTg-AD	**p=0.0076	1.530
E.Pm	non-Tg vs 3xTg-AD	**p=0.0042	1.684
p.MOI	non-Tg vs 3xTg-AD	**p=0.0046	1.689
**Bone parameters Fig.2**	**Comparison between groups**	**p value**	**Choen's d**
T.Ar	non-Tg vs 3xTg-AD	*p=0.0176	1.253
B.Ar	non-Tg vs 3xTg-AD	*p=0.0173	1.258
P.Pm	non-Tg vs 3xTg-AD	***p=0.0002	1.998
E.Pm	non-Tg vs 3xTg-AD	****p<0.0001	1.995
p.MOI	non-Tg vs 3xTg-AD	****p<0.0001	1.888
**Bone parameters Fig.3**	**Comparison between groups**	**p value**	**Choen's d**
Femur Maximum Load	non-Tg vs 3xTg-AD	*p=0.0428	1.099
Femur Stiffness	non-Tg vs 3xTg-AD	p=0.0654	0.999
Tibia Maximum Load	non-Tg vs 3xTg-AD	p=0.8937	0.0641
Tibia Stiffness	non-Tg vs 3xTg-AD	*p=0.0274	1.338
**Bone parameters Fig.4**	**Comparison between groups**	**p value**	**Choen's d**
%Empty OYs Lacunae	non-Tg vs 3xTg-AD	*p=0.0234	1.616
%TRAP+ Oys/Tot Lacunae	non-Tg vs 3xTg-AD	*p=0.0361	1.361
mRNA Dkk1	non-Tg vs 3xTg-AD	**p=0.0043	1.224
**Bone parameters Fig.5**	**Comparison between groups**	**p value**	**Choen's d**
%Ct. Nr. OCs/BS	non-Tg vs 3xTg-AD	*p=0.0474	1.275
%Tb. Nr. Ocs/BS	non-Tg vs 3xTg-AD	p=0.4351	0.601
%TRAP Surface	non-Tg vs 3xTg-AD	*p=0.0187	1.380
%Tb. TRAP Surface	non-Tg vs 3xTg-AD	p=0.9386	0.048
**Bone parameters Fig.A1**	**Comparison between groups**	**p value**	**Choen's d**
Femur BMD	non-Tg vs 3xTg-AD	p=0.6045	0.249
Femur BV/TV	non-Tg vs 3xTg-AD	p=0.1584	0.697
Femur DA	non-Tg vs 3xTg-AD	p=0.9524	0.026
Femur FD	non-Tg vs 3xTg-AD	p=0.5624	0.281
Femur Tb.N	non-Tg vs 3xTg-AD	p=0.2755	0.545
Femur Tb.Sp	non-Tg vs 3xTg-AD	p=0.1685	0.779
Femur Tb.Th	non-Tg vs 3xTg-AD	p=0.9705	0.054
Tibia BMD	non-Tg vs 3xTg-AD	p=0.0972	0.830
Tibia BV/TV	non-Tg vs 3xTg-AD	p=0.1343	0.810
Tibia DA	non-Tg vs 3xTg-AD	p=0.5894	0.262
Tibia FD	non-Tg vs 3xTg-AD	p=0.5465	0.289
Tibia Tb.N	non-Tg vs 3xTg-AD	p=0.0582	0.989
Tibia Tb.Sp	non-Tg vs 3xTg-AD	p=0.3961	0.046
Tibia Tb.Th	non-Tg vs 3xTg-AD	p=0.8976	0.062

Table S8 Effect Size. Effect sizes (Cohen's d) were computed to estimate the magnitude of the observed effects across analyses.

Figure S1

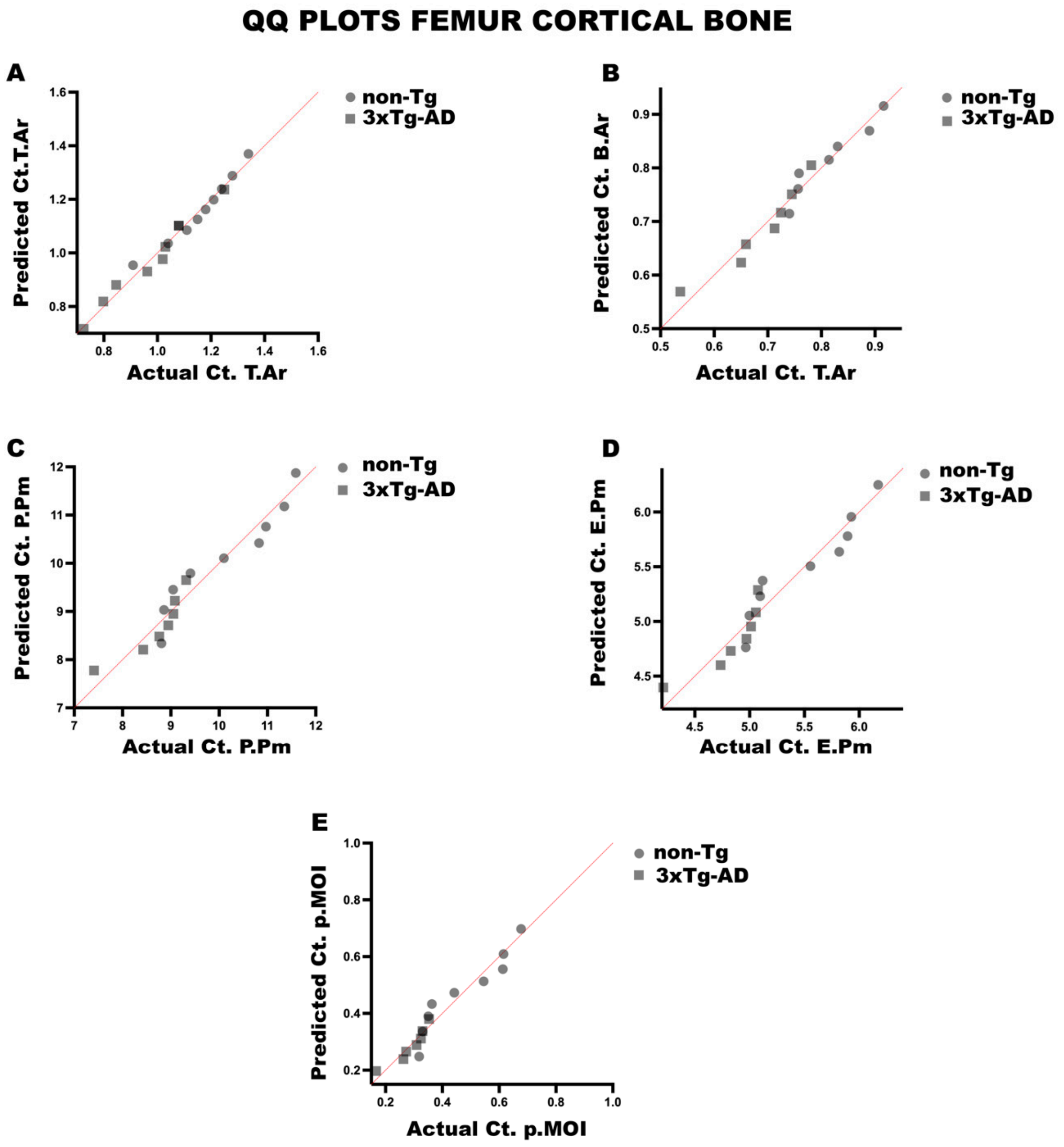

Figure S1. QQ plots femur cortical bone. The graphs show the predicted values for each measurement on the Y axis and the corresponding actual values on the X axis.

Figure S2

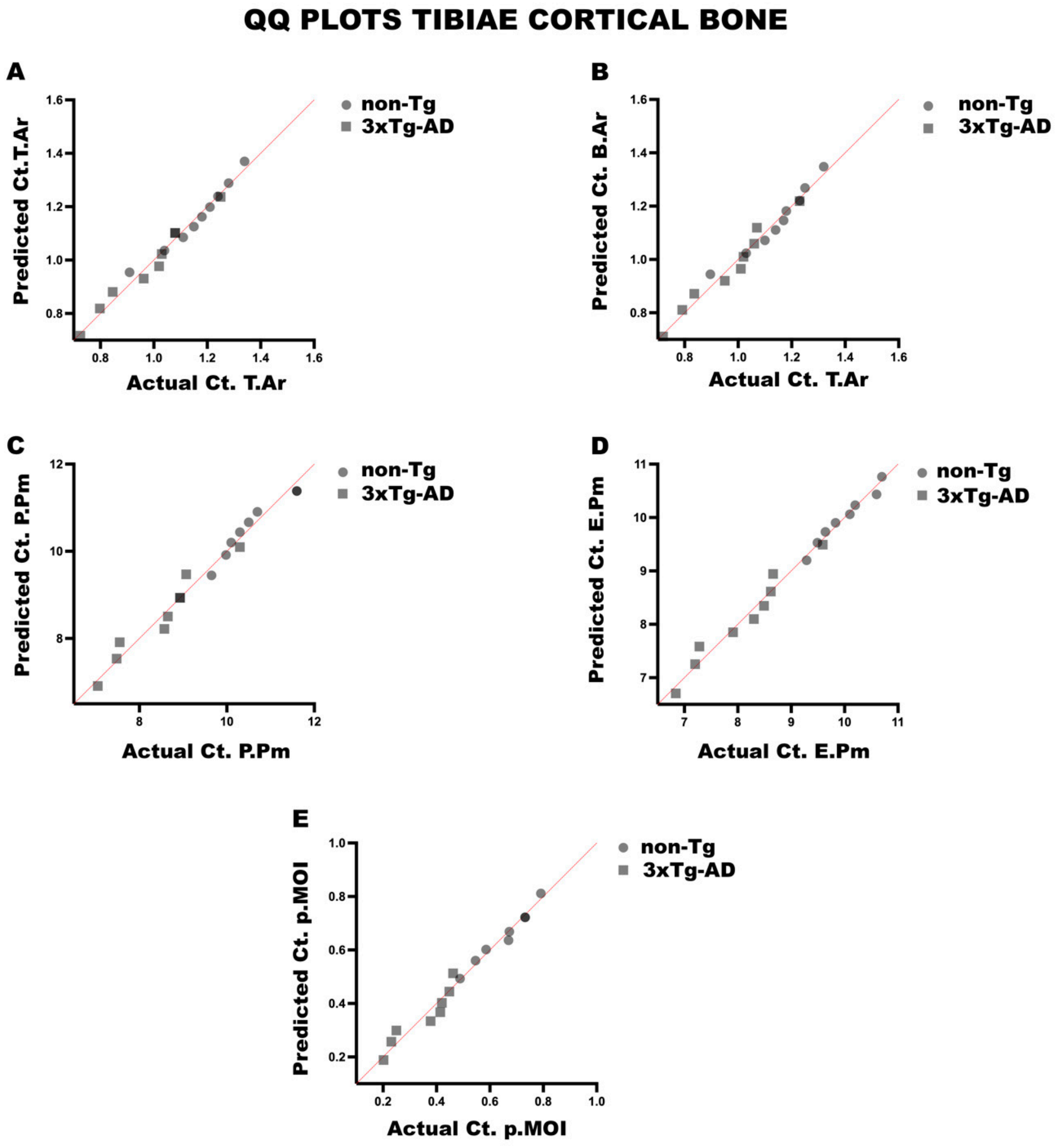

Figure S2. QQ plots tibiae cortical bone. The graphs show the predicted values for each measurement on the Y axis and the corresponding actual values on the X axis.

Figure S3

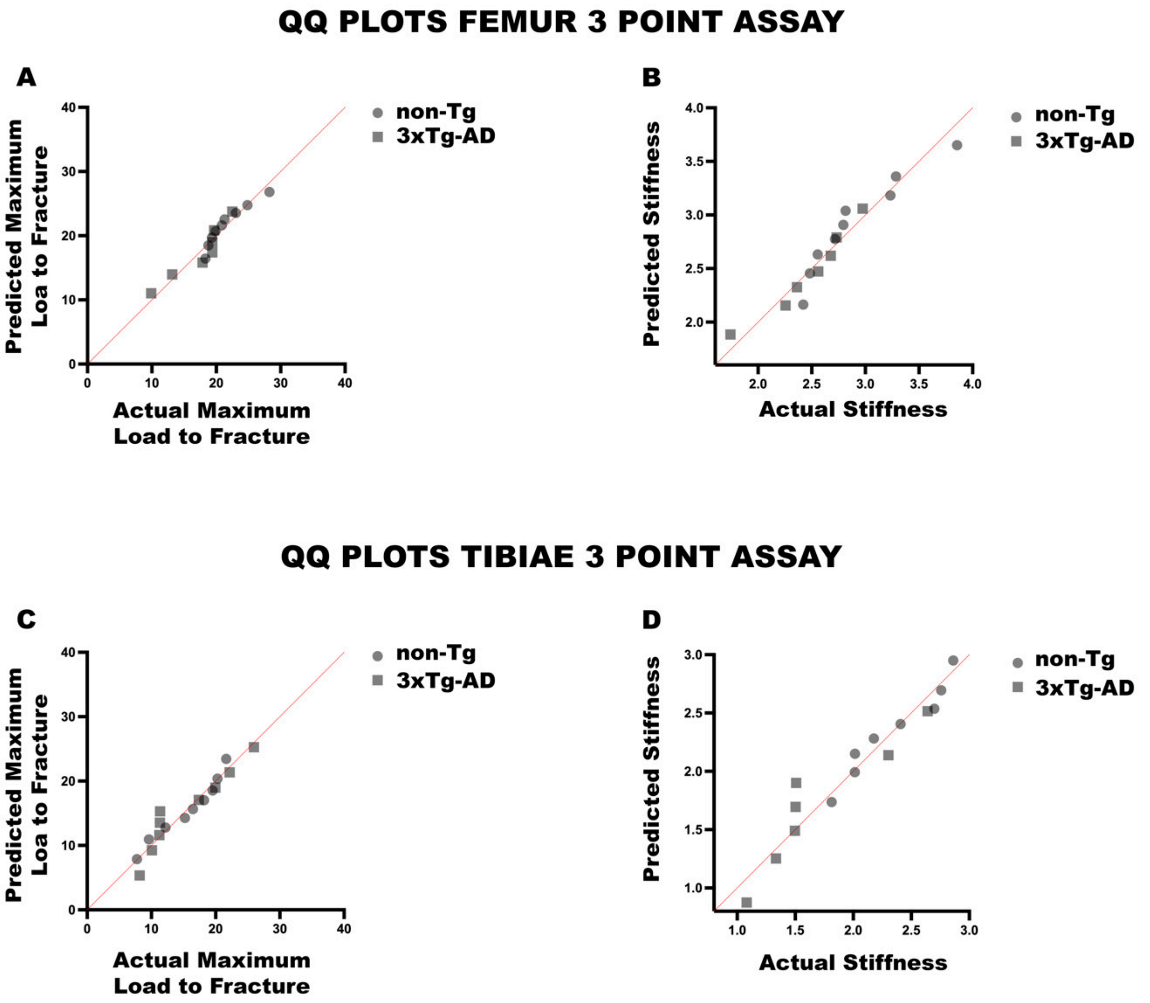

Figure S3. QQ plots 3-point test. The graphs show the predicted values for each measurement on the Y axis and the corresponding actual values on the X axis.

Figure S4

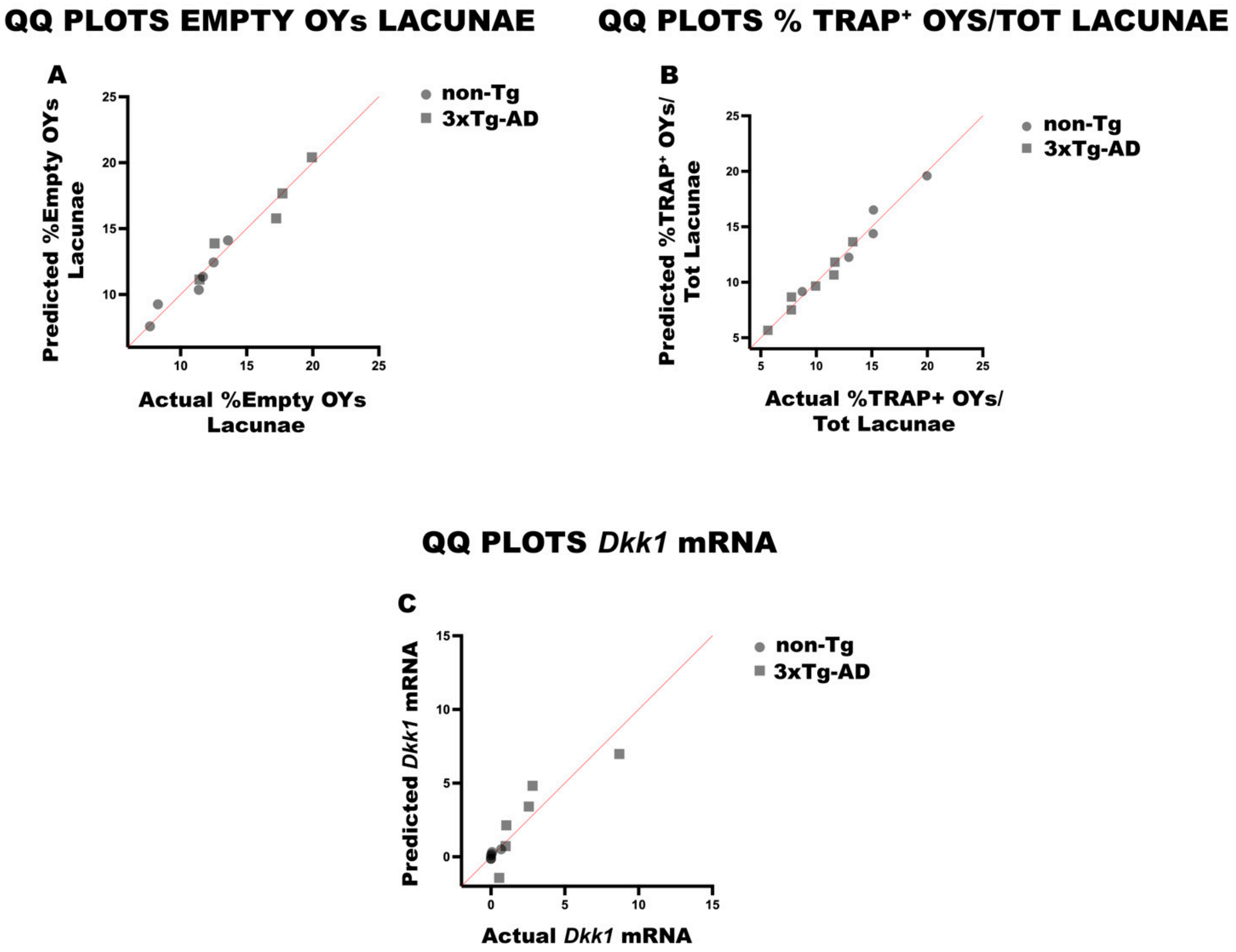

Figure S4. QQ plots OYs and *Dkk1* mRNA. The graphs show the predicted values for each measurement on the Y axis and the corresponding actual values on the X axis.

Figure S5

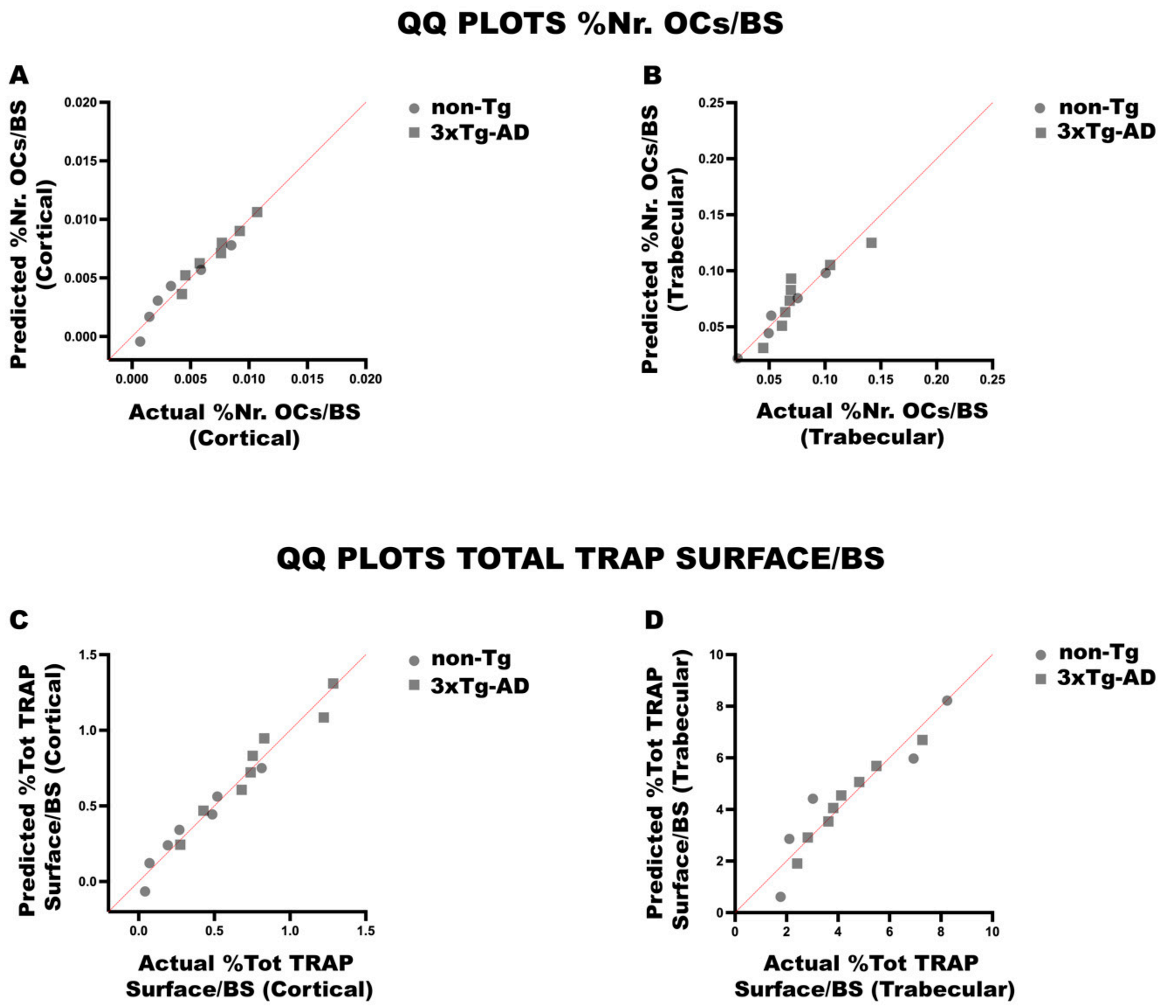

Figure S5. QQ plots OCs. The graphs show the predicted values for each measurement on the Y axis and the corresponding actual values on the X axis.

Figure S6

QQ PLOTS FEMUR TRABECULAR

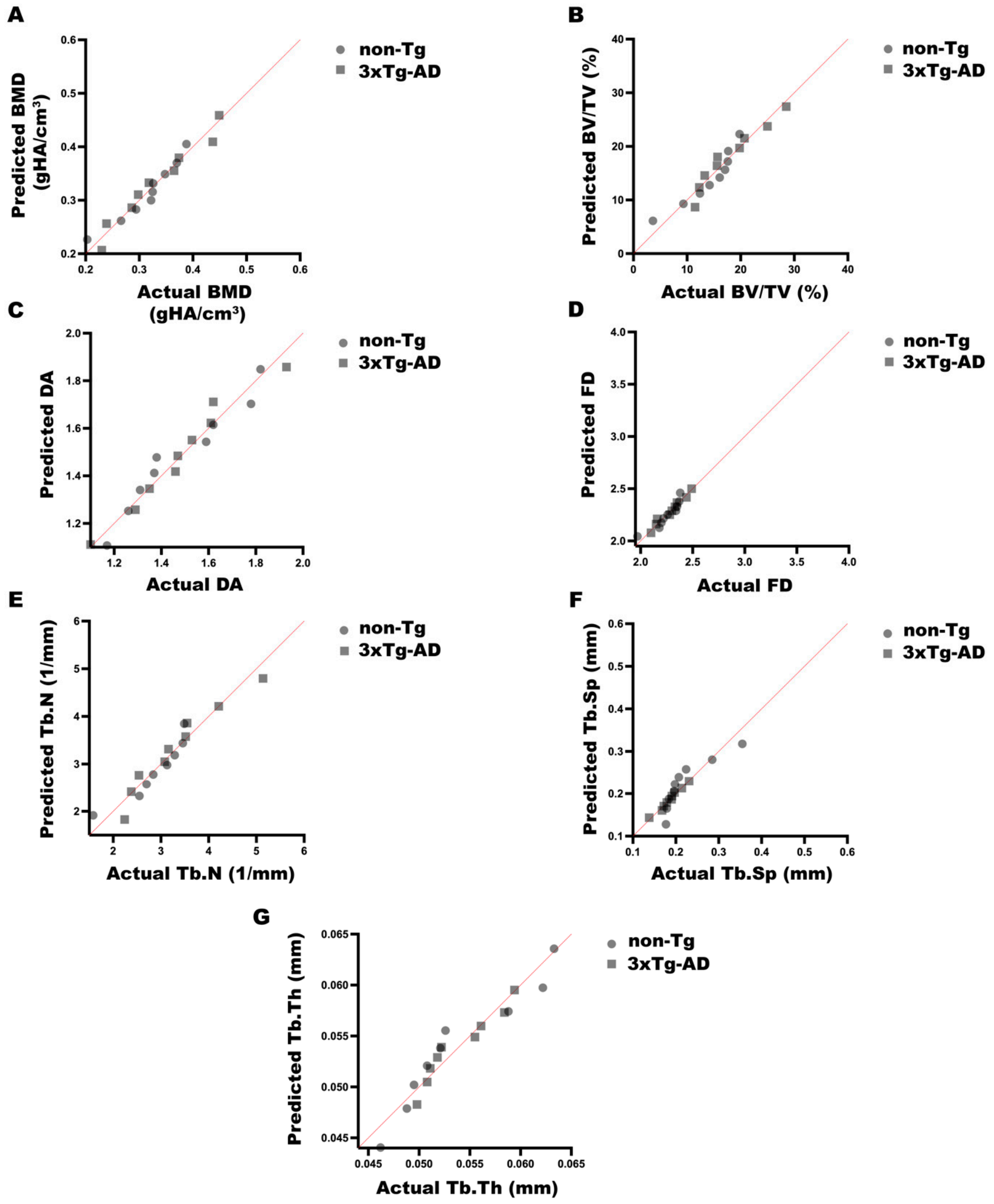

Figure S6. QQ plots femur trabecular bone. The graphs show the predicted values for each measurement on the Y axis and the corresponding actual values on the X axis.

Figure S7

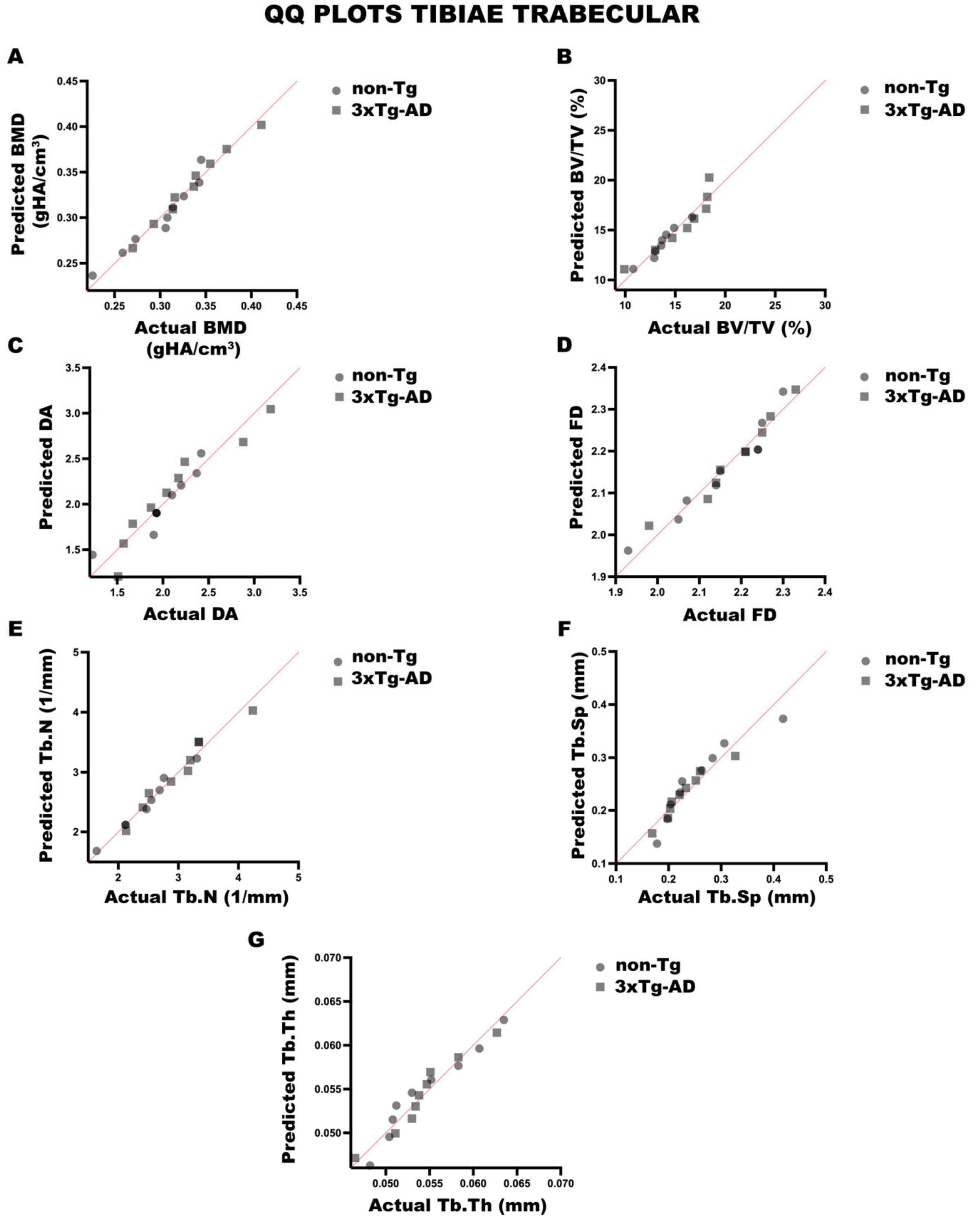

Figure S7. QQ plots tibiae trabecular bone. The graphs show the predicted values for each measurement on the Y axis and the corresponding actual values on the X axis.
